# Determining the minimal important change of the abdominal Hernia-Q

**DOI:** 10.1007/s10029-026-03645-3

**Published:** 2026-05-12

**Authors:** Salman Khan, Justus Zemberi, Timothy Olsen, Aamirah McCutchen, Malia Voytik, Sophia Jarrar, Margaret M. Hornick, Robyn B. Broach, Sumedha Chhatre, Ravishankar Jayadevappa, John P. Fischer

**Affiliations:** 1 Perelman School of Medicine, University of Pennsylvania, Philadelphia, PA USA; 2 Division of Plastic & Reconstructive Surgery, Department of Surgery, Hospital of the University of Pennsylvania, Philadelphia, PA USA; 3 Division of Plastic Surgery, Perelman Center, Hospital of the University of Pennsylvania, 14th Floor South, 3400 Spruce Street, Philadelphia, PA 1904 USA

**Keywords:** Abdominal wall reconstruction, Minimal important change, Abdominal hernia questionnaire, Hernia, Quality of life

## Abstract

**Purpose:**

The Abdominal Hernia-Q(AHQ) is a validated PRO tool for hernia patients. This study aimed to determine the Minimal Important Change (MIC) for the AHQ, examining changes in AHQ scores and exploring associations with key clinical characteristics.

**Methods:**

A retrospective cohort design was used. Patients who underwent hernia repair between January 2017 and January 2022 and completed the AHQ preoperatively and six months postoperatively were included. Anchor (Short Form Health Survey (SF-12) and Hernia-related Quality of Life Survey (HerQLes)) and distribution-based methods were used to compute the MIC. The distribution method used 0.5×standard deviation [SD] and minimal detectable change [MDC; 1.96×SEM], to identify the MIC.

**Results:**

A total of 147 patients met the inclusion criteria, with a mean age of 55.69 (SD 12.26), 60% female and a BMI of 30.8 km/m2 (26.9–36.5). A grand mean MIC of 6.8 was derived. One hundred twenty-nine patients met or exceeded the MIC, while 18 did not. Patients meeting the MIC had significantly worse baseline AHQ scores (46 vs. 68, *p* < 0.001) and higher postoperative AHQ scores (86 vs. 67, *p* < 0.001). There was no difference in operative characteristics or complications between groups, with the exception that patients not achieving MIC had a higher rate of seroma (28% vs. 9.3%, *p* = 0.038).

**Conclusion:**

The findings suggest that patients with worse preoperative status may still experience clinically significant benefits. Establishing the MIC for the AHQ may enable the identification of patients likely to experience significant improvements in quality of life after hernia repair.

**Supplementary Information:**

The online version contains supplementary material available at 10.1007/s10029-026-03645-3.

## Introduction

Each year, over 600,000 hernia repairs are performed in the United States [1]. Traditionally, surgical success has been evaluated based on technical measures such as complication rates, recurrence, and postoperative complications. While these clinical measures remain important, with healthcare’s increasing adoption of patient-centric standards of care, there is a growing emphasis on patient-reported outcomes (PROs) to capture quality of life and patient satisfaction directly from the patient’s perspective [2]. Thus, there is an urgent need for standardized assessment of patient outcomes to ensure quality and satisfaction with care [3].

The Abdominal Hernia-Q (AHQ) is a validated PRO instrument designed specifically for hernia patients to assess quality-of-life metrics, including pain, physical limitations, and emotional well-being. Developed with patient involvement, the AHQ captures a broad spectrum of abdominal wall–related symptoms and functional impacts. This may allow it to more fully reflect patient experience compared to instruments developed using clinician-generated items alone. By providing detailed insight into postoperative experiences and satisfaction, the AHQ provides a more nuanced assessment of the impact of hernia repair on their quality of life [4].

However, while the AHQ captures various domains relevant to hernia patients, it is essential to understand how to interpret the significance of change in AHQ scores, specifically the magnitude of the Minimal Important Change (MIC). The MIC represents the smallest change in score that patients perceive as meaningful, helping to differentiate between statistically significant changes and those that truly impact patient quality of life [5, 6]. Establishing an MIC for the AHQ could aid clinicians in interpreting AHQ scores in a clinically relevant way, guiding decision-making and tailoring postoperative care to better align with patient expectations [7]. This threshold could also enhance shared decision-making by enabling surgeons to discuss realistic improvement goals with patients preoperatively and evaluate the effectiveness of different surgical approaches or adjunctive interventions in achieving clinically meaningful outcomes.

In this study, we aimed to determine the MIC for the AHQ using anchor-based and distribution-based methods. We also examined postoperative AHQ scores and explored their associations with key clinical characteristics. By identifying a clinically meaningful threshold for changes in AHQ scores, this research will enhance the tool’s utility in clinical practice, helping to bridge the gap between statistical data and patient-reported outcomes in hernia repair.

## Methods

### Study design and population

Following institutional review board approval (IRB #857930), a retrospective single-institution study was conducted of patients who underwent abdominal wall reconstruction (AWR) between January 2017 and January 2022. Patients were included if they had completed both preoperative and postoperative AHQ, with the postoperative survey administered at least 180 days after surgery. Patients who experienced a hernia recurrence prior to completing their postoperative survey were excluded.

### Patient-reported outcomes

All patients received the AHQ preoperatively and postoperatively. The AHQ has eight items, and the response on each item ranges from 1 to 4. Scores were standardized to a 0–100 scale, consistent with prior literature, with higher scores indicating better quality of life [19]. For the postoperative AHQ, additional questions related to quality of care with no preoperative equivalent were excluded, including items 1b, 3a, 4a, 5a, 6a, 7a, 8a, and 9c. A subset of patients also completed the 12-Item Short Form Health Survey (SF-12) and Hernia-related Quality of Life Survey (HerQLes) at preoperative and postoperative time points. Surveys were initially administered in paper format and completed in clinic. During the study period, administration transitioned to electronic completion via REDCap on tablet devices.

## Statistical analysis

### Distribution-based MIC determination

Two distribution-based methods were used to estimate the MIC for each of the eight AHQ items and the total score. First, the standard deviation (SD) of AHQ scores at baseline (pre), follow-up (post), and change (post–pre) were used to generate MIC estimates corresponding to ½ SD and ⅓ SD thresholds. Second, the standard error of measurement (SEM) was derived using the formula SEM = SD × √(1 – Cronbach’s α), where Cronbach’s α represented internal consistency reliability and SD the measure of variability. Consistent with prior studies, a single-unit change in the SEM was interpreted as the MIC estimate for each item [8].

### Anchor-based MIC determination

Anchor-based analyses were performed in the subset of patients who completed both the AHQ and the anchor instruments (SF-12 and HerQLes), both before and after surgery. Potential anchors were screened for correlation with AHQ scores, and two items with moderate association were selected: (1) the SF-12 item 8, *“How much did pain interfere with your normal work (including both work outside the home and housework)?”*, and (2) the HerQLes item 9, *“I accomplish less at home because of my abdominal wall.”* These anchors were used to estimate AHQ MICs by examining the relationship between changes in anchor responses and changes in AHQ scores.

### Final MIC estimate

For each item on the AHQ and the total score, both distribution-based (½ SD, 1/3 SD, and standard error of measurement) and anchor-based MICs were derived using AHQ scores standardized to a 0–100 scale derived from matched pre- and postoperative items. Standardization was performed to facilitate interpretability and consistency with prior AHQ literature. The grand mean MIC was calculated by averaging the estimates from both approaches.

Once the final MIC threshold was established, patients were dichotomized into two groups: those who met or exceeded the MIC and those who did not. Demographic and clinical variables were compared between groups using appropriate statistical tests, including the chi-square test for categorical variables and the t-test or Mann–Whitney U test for continuous variables. Statistical significance was defined as *p* < 0.05. All analyses were conducted using R program (version 4.3.3).

## Results

### Study cohort

The cohort consisted of 147 patients who underwent hernia repair between January 2017 and January 2022 and completed the AHQ preoperatively and six months postoperatively. Baseline demographics and clinical characteristics are summarized in Table 1.

**Table 1:** Patient demographics

	All (n=147)^1^
Age (years)	55.69 (12.26)
Gender	
Male	60 (4)
Female	87 (60)
Race	
African American	23 (15)
White	114 (78)
Asian	1 (1)
Unknown	9 (6)
Ethnicity	
Non-Latino or Non-Hispanic	142 (97)
Latino or Hispanic	3 (2)
Unknown	2 (1)
History of	
Cancer	24 (17)
COPD	9 (6)
Diabetes	12 (86)
Hypertension	69 (47)
PVD	5 (3)
Smoking	
Never	76 (52)
Current	14 (10)
Former (quit for >30days)	57 (39)

^1^n (%); Mean (SD)

### AHQ scores

At baseline, the mean total AHQ score was 48.78 (Cronbach’s alpha = 0.89). At six months postoperatively, the mean total AHQ score was 84.10 (Cronbach’s alpha = 0.90). The distributions of baseline and postoperative AHQ scores are shown in Tables 3 and 2, respectively.

**Table 2:** Abdominal Hernia-Q (AHQ) > 180 days postoperative scores

	Mean	Standard Deviation	Confidence Interval	Internal Consistency Reliability*
In the last two weeks, how often have you experienced the following:				
In the last two weeks, I have had severe pain related to my hernia	87.75	23.76	83.88, 91.63	0.92
In the last two weeks, my hernia has made my sleep worse	87.98	22.05	84.39, 91.58	0.91
In the last two weeks, my hernia has made it harder to do my daily routine (e.g. what I do from the time I get up, until I go to bed)	84.13	25.08	80.04, 88.21	0.91
In the last two weeks, my hernia has limited how much I can get done by myself (e.g. without someone to help me	83.44	26.28	79.16, 87.73	0.92
In the last two weeks, my hernia has made me feel anxious	87.30	23.84	83.41, 91.19	0.92
In the last two weeks, my hernia has made me feel less attractive without my clothes on	80.27	30.41	75.31, 85.23	0.92
The symmetry (evenness) of my abdomen	78.23	26.36	73.93, 82.53	0.93
How normal I feel in my clothing with my hernia	83.67	26.57	79.34, 88.00	0.92
**Total Score**	84.10	19.48	80.92, 87.27	0.90

* Cronbach’s alpha

**Table 3:** Abdominal Hernia-Q (AHQ) preoperative scores

	Mean	Standard Deviation	Confidence Interval	Internal Consistency Reliability*
In the last two weeks, how often have you experienced the following:				
In the last two weeks, I have had severe pain related to my hernia	67.34	34.34	61.75, 72.94	0.91
In the last two weeks, my hernia has made my sleep worse	68.02	34.43	62.41, 25.79	0.91
In the last two weeks, my hernia has made it harder to do my daily routine (e.g. what I do from the time I get up, until I go to bed)	58.73	36.25	52.82, 73.64	0.90
In the last two weeks, my hernia has limited how much I can get done by myself (e.g. without someone to help me	68.02	33.76	62.52, 64.64	0.91
In the last two weeks, my hernia has made me feel anxious	56.01	38.22	49.78, 73.53	0.91
In the last two weeks, my hernia has made me feel less attractive without my clothes on	35.37	39.06	29.01, 62.24	0.91
The symmetry (evenness) of my abdomen	15.87	26.27	11.59, 41.74	0.91
How normal I feel in my clothing with my hernia	20.86	30.27	15.93, 20.15	0.91
**Total Score**	**48.78**	**25.55**	**44.61**,** 52.94**	**0.89**

* Cronbach’s alpha

### Distribution-based MIC

Using the standard deviation method, the 1/2 SD estimate for the difference in AHQ score (post – pre) was 12.5, the 1/3 SD estimate was 8.3, and the standard error of measurement was 9.9. MIC estimates for each individual AHQ item preoperatively, postoperatively, the difference (post – pre), and the average of all three are presented in Table 4.

**Table 4:** MIC Calculation

	Distribution Method												Anchor Method		Grand Mean MIC
	A			B			C			D			Pre	Pre	
	Pre			Post			Post-Pre			Average of A, B, and C					
	1/2SD	1/3SD	1SEM	1/2SD	1/3SD	1SEM	1/2SD	1/3SD	1SEM	1/2SD	1/3SD	1SEM	SF12Item 8	HerQLesItem 9	
In the last two weeks, how often have you experienced the following:															
In the last two weeks,I have had severe pain related to my hernia	10.3	17.1	11.5	11.9	7.9	6.7	17.4	11.6	12.1	13.2	12.2	10.1	19.3	8.4	12.8
In the last two weeks, how often has your hernia affected the following:															
In the last two weeks, my hernia has made my sleep worse	17.2	11.5	10.3	11.0	7.4	6.6	16.4	10.9	14.7	14.9	9.9	10.5	22.4	6.5	13.1
In the last two weeks, my hernia has made it harder to do my daily routine (e.g. what I do from the time I get up, until I go to bed)	18.1	12.1	11.5	12.5	8.4	7.5	17.9	11.9	13.4	16.2	10.8	10.8	6.8	10.0	10.7
In the last two weeks, my hernia has limited how much I can get done by myself (without someone to help me)	16.9	11.3	10.1	13.1	8.8	7.9	17.2	11.4	12.4	15.7	10.5	10.1	18.6	11.5	13.6
In the last two weeks, my hernia has made me feel anxious	19.1	12.7	11.5	11.9	7.9	6.7	19.1	12.8	13.8	16.7	11.2	10.7	1.8	7.5	8.8
In the last two weeks, my hernia has made me feel less attractive without my clothes on	19.5	13.0	11.7	15.2	10.1	8.6	21.1	14.1	14.6	18.6	12.4	11.6	4.0	10.8	10.8
The symmetry (evenness) of my abdomen	13.1	8.8	7.9	13.2	8.8	6.9	18.1	12.1	12.6	14.8	9.9	9.1	11.3	7.3	10.3
How normal I feel in my clothing with my hernia	15.1	10.1	9.1	13.3	8.9	7.5	18.9	12.6	13.6	15.8	10.5	10.1	6.3	4.1	8.7
Total Score	12.8	8.5	8.5	9.8	6.5	6.2	12.5	8.3	9.9	11.7	7.8	8.2	6.0	2.8	6.8

### Anchor-based MIC

A subset of 73 patients also completed both the SF-12 and the HerQLes. Both of the selected anchors demonstrated moderate correlations with baseline AHQ scores. Anchor-based analyses yielded MIC estimates of 6.0 for SF-12 item eight and 2.8 for HerQLes item nine. Individual anchor-based MIC estimates for each AHQ item are presented in Table 4.

### Final MIC

The grand mean MIC, calculated by averaging distribution- and anchor-based estimates, was 6.8 points for the total AHQ score on the standardized 0–100 scale. For reference, this corresponds to approximately 9.6 points on the 8–32 total score scale and 1.2 points on the original 1–4 item response scale. Item-level grand mean MIC values are also presented in Table 4.

### Comparison of patients achieving versus not achieving MIC

Patients were stratified into two groups based on whether they achieved an improvement greater than or equal to the MIC threshold for the grand mean MIC (6.8). A total of 129 patients (87.8%) met or surpassed the grand mean MIC (6.8), while 18 patients (12.2%) did not.

Baseline demographics were similar between the two groups (Table 5). There was no significant difference in gender, BMI, race, American Society of Anesthesiologists (ASA) score, smoking history, ventral hernia working group (VHWG) grade, diabetes history, or COPD. However, the demographics associated with surpassing the MIC were a lower preoperative AHQ score (*p* < 0.001), a higher postoperative AHQ score (*p* = 0.005), and an older age at surgery (*p* = 0.005).

**Table 5:** Demographic characteristics of patientsachieving and not achieving MIC

	Response Category			
	Variable	< MICN = 18^1^	> MICN = 129^1^	p-value^2^
Pre AHQ score	68 (29)	46 (24)	< 0.001	
Post AHQ score	67 (28)	86 (18)	< 0.001	
Age	47 (11)	57 (12)	0.005	
Gender			0.061	
Male	11 (61)	49 (38)		
Female	7 (39)	80 (62)		
BMI	32 (5)	32 (7)	0.4	
Race			0.063	
Black	6 (33)	17 (13)		
White	10 (56)	104 (81)		
Asian	0 (0)	1 (0.8)		
Unknown	2 (11)	7 (5.4)		
ASA Score			0.6	
1	0 (0%)	5 (3.9)		
2	11 (61%)	59 (46)		
3	7 (39)	64 (50)		
4	0 (0)	1 (0.8)		
Smoking History	5 (28)	66 (51)	0.063	
Ventral Hernia Working Group Grade			0.4	
1	4 (22)	38 (29)		
2	13 (72)	69 (53)		
3	1 (6)	21 (16)		
4	0 (0)	1 (1)		
Diabetes History	0 (0)	21 (16)	0.076	
COPD	1 (6)	8 (6)	> 0.9	

^1^Mean (SD); n (%)

^2^Wilcoxon rank sum test; Pearson’s Chi-squared test; Fisher’s exact test

There were no differences in any surgical characteristics between patients who surpassed MIC and those who did not, including postoperative wound class, defect width, concurrent panniculectomy, number of previous hernia repairs, and number of previous abdominal surgeries (Table 6).

**Table 6:** Surgical characteristics of patients achieving and not achieving MIC

	Response Category			
	Variable	< MIC N = 18^1^	> MIC N = 129^1^	p-value^2^
Postoperative Wound Class:			0.5	
Clean	15 (83)	106 (82%)		
Clean-Contaminated	2 (11)	20 (16%)		
Contaminated	0 (0)	1 (1)		
Dirty-Infected	1 (6)	1 (1)		
Unknown	0 (0)	1 (1)		
Defect Width (cm)	11 (8)	14 (15)	0.5	
Missing	3 (17)	22 (17)		
Concurrent Panniculectomy	5 (28)	37 (29)	> 0.9	
Number of Previous Hernia Repair	0.67 (0.84)	0.81 (1.40)	0.8	
Number of Previous Abdominal Surgeries	2.44 (1.65)	3.37 (2.54)	.2	

^1^n (%); Mean (SD)

^2^Fisher’s exact test; Wilcoxon rank sum test; Pearson’s Chi-squared test

Finally, postoperative outcomes were similar between the two groups. There was no difference in rates of cellulitis, surgical site infection, delayed wound healing, wound dehiscence, mesh infection, enterocutaneous (EC) fistula, postoperative bowel obstruction, ileus, hernia recurrence, reoperation, readmission, or ED visits. However, those who surpassed MIC had significantly lower rates of postoperative seroma (*p* = 0.038) (Table 7).

**Table 7:** Surgical outcomes of patients achieving and not achieving MIC

Outcome	0 *N* = 18^1^	1 *N* = 129^1^	*p*-value^2^
Cellulitis	1 (5.6)	11 (8.5)	> 0.9
Surgical Site Infection	2 (11)	15 (12)	> 0.9
Seroma	5 (28)	12 (9.3)	0.038
Delayed Healing	5 (28)	25 (19)	0.5
Wound Dehiscence	1 (5.6)	5 (3.9)	0.5
Mesh Infection	0 (0)	1 (0.8)	> 0.9
Enterocutaneous Fistula	1 (5.6)	0 (0)	0.12
Postoperative Bowel Obstruction	1 (5.6)	4 (3.1)	0.5
Ileus	0 (0)	3 (2.3)	> 0.9
Hernia Recurrence	2 (11)	15 (12)	0.7
Reoperation	3 (17)	26 (20)	> 0.9
Readmission	3 (17)	26 (20)	> 0.9
ED Visits	5 (28)	30 (23)	0.8

^1^n (%)

^2^Fisher’s exact test

## Discussion

This study establishes a clinically meaningful threshold for change in the AHQ among patients undergoing elective abdominal wall reconstruction. Using a combination of anchor-based and distribution-based methods, we identified a 6.8-point improvement on a standardized 0–100 AHQ scale as the MIC perceived by patients. Patients who surpassed this threshold had significantly lower baseline AHQ scores, were older at the time of surgery, and experienced lower rates of postoperative seroma formation.

Our finding that patients with worse preoperative AHQ scores were significantly more likely to achieve a clinically meaningful improvement aligns with prior hernia-specific quality-of-life literature [9]. Using data from the Abdominal Core Health Quality Collaborative (ACHQC), Holla et al. demonstrated that frail patients reported the greatest improvement in quality of life one year after hernia repair, although a formal MIC was not established [10]. Frailty is closely associated with greater baseline functional limitation and symptom burden, providing increased opportunity for measurable postoperative improvement. Similarly, Renshaw et al., using the HerQLes instrument, found that patients with lower baseline quality of life, larger hernia defects, and higher ASA class were more likely to surpass the MIC [11]. Collectively, these findings suggest that baseline symptom burden strongly influences the magnitude of patient-perceived benefit following hernia repair.

This body of literature supports the use of the AHQ not only as a postoperative assessment tool, but also as a means of contextualizing expected benefit during preoperative counseling. Identifying patients with substantial baseline impairment may help surgeons better communicate anticipated improvements and address hesitancy toward operative intervention. Importantly, these findings do not suggest that patients with higher baseline quality of life fail to benefit from surgery, but rather that the magnitude of measurable improvement captured by patient-reported outcome instruments may be greatest among those with more severe preoperative impairment.

Beyond baseline quality-of-life status and age, we did not observe associations between achieving the MIC and operative complexity, prior surgical history, or most postoperative complications. These findings suggest that meaningful improvements in hernia-related quality of life may occur independently of traditional surgical risk factors and reinforce the concept that patient-reported recovery represents a distinct dimension of outcome, complementary to but not fully explained by morbidity metrics such as reoperation or readmission. This distinction is increasingly relevant as value-based surgical care emphasizes patient experience alongside technical success.

Seroma formation was the only postoperative event that differed significantly between patients who did and did not achieve the MIC. Large cohort studies from the ACHQC have demonstrated that differences in wound morbidity following abdominal wall reconstruction are frequently driven by seroma formation, although these events are often classified as low severity and managed nonoperatively [12–14]. While seromas are traditionally viewed as benign in the absence of infection or reintervention, they may contribute to prolonged discomfort, activity limitation, anxiety, or dissatisfaction that is not fully captured by conventional clinical complication metrics.

Prior literature has reported no difference in absolute postoperative quality-of-life scores between patients with and without seromas [15]. However, such analyses typically rely on cross-sectional postoperative assessments, whereas the present study evaluates within-patient change relative to baseline. As a result, seroma formation may not substantially lower final quality-of-life scores, yet may attenuate the magnitude of postoperative improvement, reducing the likelihood that patients perceive their recovery as clinically meaningful. This distinction suggests that seromas may influence the recovery trajectory rather than the final health state and highlights the value of MIC-based analyses in detecting patient-centered effects that may be obscured by absolute score comparisons.

Although prior studies have established MIC thresholds for other hernia-related instruments, defining an AHQ-specific MIC remains important. Neela et al. used an anchor-based approach to estimate a HerQLes MIC of 7 points; however, their cohort included patients without ventral hernias, and only six patients ultimately underwent hernia repair. Consequently, the study provides limited insight into the impact of hernia repair on postoperative quality-of-life improvement and is not directly applicable to determining a clinically meaningful change in surgical patients [16]. Additionally, Renshaw et al.’s HerQLes analysis relied solely on distribution-based estimates. In contrast, our study integrates both distribution-based and anchor-based approaches, allowing for a more clinically grounded interpretation of meaningful change. Anchor-based methods directly relate changes in AHQ scores to patient-reported functional interference and activity limitation, strengthening the clinical validity of the MIC estimate [17, 18]. Establishing a unique AHQ MIC is particularly valuable given that the AHQ was developed with direct patient involvement and captures a broad spectrum of abdominal wall–related symptoms and functional impacts. Prior studies demonstrating the AHQ’s reliability, responsiveness, and construct validity further support the relevance of defining an AHQ-specific MIC [4].

Determining a clinically meaningful threshold for AHQ improvement has several implications for patient-centered care. First, an established MIC can help surgeons contextualize postoperative AHQ scores and evaluate whether observed changes are meaningful from the patient’s perspective. Second, integrating MIC thresholds into preoperative counseling may enhance shared decision-making by allowing clinicians to provide realistic expectations regarding quality-of-life improvement. Third, because our findings suggest that patients with worse baseline status may derive greater benefit, the AHQ MIC may help guide discussions around surgical candidacy, particularly in older or symptomatic patients in whom the balance of risk and potential benefit must be carefully weighed.

Despite the strengths of this study, several limitations should be acknowledged. First, only a subset of patients completed the SF-12 and HerQLes instruments, which reduced the sample size available for anchor-based analyses and may have limited the precision of those estimates. Second, although all postoperative AHQ surveys were completed at least six months after surgery, ensuring sufficient time for recovery, the exact timing of survey administration was not standardized, introducing potential variability in postoperative response patterns. Prior studies assessing quality-of-life changes after hernia repair have demonstrated stabilization of PROs by six months postoperatively, with no significant differences between 3 and 6-month assessments, supporting the use of this timepoint to capture meaningful recovery [19]. Despite these limitations, the use of both anchor-based and distribution-based methods, the use of a validated hernia-specific PRO instrument, and a large, single-institution cohort with consistent pre- and postoperative AHQ collection strengthen the overall reliability and clinical relevance of the findings. Finally, to further validate the methods and findings related to the AHQ MIC, larger prospective studies with more complete SF-12 data collection to enhance the statistical power of the anchor-based analysis and reduce bias from missing data.

## Conclusion

This study establishes the MIC for the AHQ in patients undergoing elective AWR, identifying a 6.8-point improvement in the total score as clinically meaningful on a standardized 0–100 scale. Patients with lower baseline AHQ score and older age were more likely to exceed this threshold. Seroma formation was more common among patients who did not surpass the MIC, suggesting that seromas may have a greater impact on postoperative quality of life than previously appreciated. Future prospective studies with larger cohorts and more complete anchor data are needed to further validate these findings and clarify the significance of seroma formation in relation to PROs.

## Supplementary Information

Below is the link to the electronic supplementary material.

Supplementary Material 1

Supplementary Material 2

Supplementary Material 3

Supplementary Material 4

Supplementary Material 5

Supplementary Material 6

Supplementary Material 7

Supplementary Material 8

Supplementary Material 9

Supplementary Material 10

Supplementary Material 11

## Data Availability

A de-identified version of the datasets generated during and/or analyzed during the current study is available from the corresponding author on reasonable request.

## References

[1] Schlosser KA, Renshaw SM, Tamer RM et al (2023) Ventral hernia repair: an increasing burden affecting abdominal core health. Hernia 27:415–421. 10.1007/s10029-022-02707-636571666 10.1007/s10029-022-02707-6

[2] Krpata DM, Schmotzer BJ, Flocke S, Jin J, Blatnik JA, Ermlich B, Novitsky YW, Rosen MJ (2012) Design and initial implementation of HerQLes: a hernia-related quality-of-life survey to assess abdominal wall function. J Am Coll Surg 215:635–642. 10.1016/j.jamcollsurg.2012.06.41222867715 10.1016/j.jamcollsurg.2012.06.412

[3] Huerta S, Varshney A, Patel PM, Mayo HG, Livingston EH (2016) Biological mesh implants for abdominal hernia repair. JAMA Surg 151:374. 10.1001/jamasurg.2015.523426819222 10.1001/jamasurg.2015.5234

[4] Patel V, Cunning JR, Rios-Diaz AJ, Mauch JT, Nathan SL, Messa CA, Whitely CB, Kozak GM, Broach RB, Fischer JP (2020) Prospective assessment of the abdominal hernia-Q (AHQ): patient burden, reliability, and longitudinal assessment of quality of life in hernia repair. Ann Surg 276:1039–1046. 10.1097/SLA.000000000000471333630470 10.1097/SLA.0000000000004713PMC9645545

[5] Jayadevappa R, Cook R, Chhatre S (2017) Minimal important difference to infer changes in health-related quality of life: a systematic review. J Clin Epidemiol 89:188–198. 10.1016/j.jclinepi.2017.06.00928676426 10.1016/j.jclinepi.2017.06.009

[6] Crosby RD, Kolotkin RL, Williams GR (2003) Defining clinically meaningful change in health-related quality of life. J Clin Epidemiol 56:395–407. 10.1016/S0895-4356(03)00044-112812812 10.1016/s0895-4356(03)00044-1

[7] Patel V, Jia H, Rios-Diaz AJ, Christopher AN, Morris MP, Diatta F, Cunning JR, Broach RB, Fischer JP (2022) Law of diminishing returns in ventral hernia repair: fact or fiction? Plast Reconstr Surg 149:964–972. 10.1097/PRS.000000000000895135188905 10.1097/PRS.0000000000008951

[8] Wyrwich KW, Tierney WM, Wolinsky FD (1999) Further evidence supporting an SEM-based criterion for identifying meaningful intra-individual changes in health-related quality of life. J Clin Epidemiol 52:861–873. 10.1016/S0895-4356(99)00071-210529027 10.1016/s0895-4356(99)00071-2

[9] Licari L, Guercio G, Campanella S, Scerrino G, Bonventre S, Tutino R, Gulotta L, Profita G, Scaturro D, Mauro GL, Salamone G (2019) Clinical and functional outcome after abdominal wall incisional hernia repair: evaluation of quality-of-life improvement and comparison of assessment scales. World J Surg 43:1914–1920. 10.1007/S00268-019-05003-031011821 10.1007/s00268-019-05003-0

[10] Renshaw SM, Gupta A, Poulose BK (2022) Establishing the minimal clinically important difference for the hernia-related quality of life survey (HerQLes). Am J Surg 223:245–249. 10.1016/j.amjsurg.2021.06.01834256930 10.1016/j.amjsurg.2021.06.018

[11] Holla S, Renshaw S, Olson M, Whalen A, Sreevalsan K, Poulose BK, Collins CE (2024) Quality of life among older patients after elective ventral hernia repair: a retrospective review. Surgery 175:1547–1553. 10.1016/j.surg.2024.02.00238472081 10.1016/j.surg.2024.02.002

[12] Kim JE, Mourad M, Phillips SE et al (2024) Association of changes in HerQLes scores with objective hernia outcomes: an analysis of the ACHQC database. Surg Endosc 38:6812–6826. 10.1007/s00464-024-11140-y39164436 10.1007/s00464-024-11140-y

[13] Petro CC, Haskins IN, Tastaldi L, Tu C, Krpata DM, Rosen MJ, Prabhu AS (2018) Does active smoking really matter before ventral hernia repair? Surg 165:406–411. 10.1016/j.surg.2018.07.03910.1016/j.surg.2018.07.03930220485

[14] Haskins IN, Krpata DM, Prabhu AS, Tastaldi L, Perez AJ, Tu C, Rosenblatt S, Poulose BK, Rosen MJ (2018) Immunosuppression is not a risk factor for 30-day wound events or additional 30-day morbidity or mortality after open ventral hernia repair. Surg 164:594–600. 10.1016/j.surg.2018.05.02310.1016/j.surg.2018.05.02330029991

[15] Miller BT, Baier KF, Zolin SJ et al (2023) Long-term outcomes of seromas after ventral hernia repair: a propensity score-matched analysis of the Abdominal Core Health Quality Collaborative. Hernia 27:373–378. 10.1007/s10029-022-02613-x35437694 10.1007/s10029-022-02613-x

[16] Neela N, Olavarria OA, Rondon AP, Bernardi K, Shah P, Dhanani N, Lyons N, Matta EJ, Hasapes JP, Liang MK (2022) Validation of the minimal clinically important difference for modified Activities Assessment Scale. Am J Surg 223:770–773. 10.1016/j.amjsurg.2021.07.04234325909 10.1016/j.amjsurg.2021.07.042

[17] Barrett B, Brown RL, Mundt MP (2008) Comparison of anchor-based and distributional approaches in estimating important difference in common cold. Qual Life Res 17:75–85. 10.1007/S11136-007-9277-218027107 10.1007/s11136-007-9277-2

[18] Jayadevappa R, Malkowicz SB, Wittink MN, Wein AJ, Chhatre S (2012) Comparison of distribution- and anchor-based approaches to infer changes in health-related quality of life of prostate cancer survivors. Health Serv Res 47:1902–1925. 10.1111/j.1475-6773.2012.01395.x22417225 10.1111/j.1475-6773.2012.01395.xPMC3513611

[19] Lawrence K, McWhinnie D, Jenkinson C, Coulter A (1997) Quality of life in patients undergoing inguinal hernia repair. Ann R Coll Surg Engl 79:40–459038494 PMC2502599

